# Risk of dyslipidemia and major adverse cardiac events with tofacitinib *versus* adalimumab in rheumatoid arthritis: a real-world cohort study from 7580 patients

**DOI:** 10.3389/fphar.2024.1370661

**Published:** 2024-05-31

**Authors:** Xiao-Na Ma, Mei-Feng Shi, Shiow-Ing Wang, Wei Feng, Shu-Lin Chen, Xiao-Qin Zhong, Qing-Ping Liu, James Cheng-Chung Wei, Chang-Song Lin, Qiang Xu

**Affiliations:** ^1^ State Key Laboratory of Traditional Chinese Medicine Syndrome, The First Clinical Medical College of Guangzhou University of Chinese Medicine, Guangzhou, China; ^2^ Department of Rheumatology, The First Affiliated Hospital of Guangzhou University of Chinese Medicine, Guangzhou, China; ^3^ Center for Health Data Science, Department of Medical Research, Chung Shan Medical University Hospital, Taichung, Taiwan; ^4^ Institute of Medicine, Chung Shan Medical University, Taichung, Taiwan; ^5^ Department of Allergy, Immunology & Rheumatology, Chung Shan Medical University Hospital, Taichung, Taiwan; ^6^ Department of Nursing, Chung Shan Medical University, Taichung, Taiwan; ^7^ Graduate Institute of Integrated Medicine, China Medical University, Taichung, Taiwan; ^8^ Office of Research and Development, Asia University, Taichung, Taiwan

**Keywords:** tofacitinib, adalimumab, lipidemias, risk factors, TriNetX

## Abstract

**Objective:**

To compare the effects of tofacitinib and adalimumab on the risk of adverse lipidaemia outcomes in patients with newly diagnosed rheumatoid arthritis (RA).

**Methods:**

Data of adult patients newly diagnosed with RA who were treated with tofacitinib or adalimumab at least twice during a 3-year period from 1 January 2018 to 31 December 2020, were enrolled in the TriNetX US Collaborative Network. Patient demographics, comorbidities, medications, and laboratory data were matched by propensity score at baseline. Outcome measurements include incidental risk of dyslipidemia, major adverse cardiac events (MACE) and all-cause mortality.

**Results:**

A total of 7,580 newly diagnosed patients with RA (1998 receiving tofacitinib, 5,582 receiving adalimumab) were screened. After propensity score matching, the risk of dyslipidaemia outcomes were higher in the tofacitinib cohort, compared with adalimumab cohort (hazard ratio [HR] with 95% confidence interval [CI], 1.250 [1.076–1.453]). However, there is no statistically significant differences between two cohorts on MACE (HR, 0.995 [0.760–1.303]) and all-cause mortality (HR, 1.402 [0.887–2.215]).

**Conclusion:**

Tofacitinib use in patients with RA may increase the risk of dyslipidaemia to some extent compared to adalimumab. However, there is no differences on MACE and all-cause mortality.

## 1 Introduction

Rheumatoid arthritis (RA) is a chronic systemic inflammatory autoimmune disease that requires long-term treatment to suppress inflammation and prevent progressive joint damage. Two of the most commonly prescribed RA medications with differing mechanisms are tofacitinib and adalimumab (Zullow et al., 2014; Zubkov et al., 2021; Xu et al., 2022; Eqbal et al., 2023; Li et al., 2023). Tofacitinib is an oral small molecule Janus kinase (JAK) inhibitor that interferes with inflammatory cytokine signaling pathways (Zhu et al., 2021). In contrast, adalimumab is an injectable tumor necrosis factor-alpha (TNF-α) inhibitor monoclonal antibody biologic that directly targets inflammatory cells and cytokines (Zullow et al., 2014; Panaccione et al., 2021; Huang et al., 2022; Zouboulis et al., 2023). Both drugs are widely used either alone or in combination strategies to manage RA symptoms and slow disease progression, often in side-by-side comparative studies (Zhang et al., 2014; Fleischmann et al., 2017; Deakin et al., 2023).

Prior studies show somewhat conflicting results on how treatment with tofacitinib *versus* adalimumab differentially affects blood lipid levels, which can elevate cardiovascular disease risk if abnormal. Some systematic reviews and meta-analyses associate tofacitinib treatment with elevations in serum cholesterol, low-density lipoprotein (LDL), and high-density lipoprotein (HDL) levels that appear independent of dosage, while TNF-α antagonists like adalimumab have no significant lipid effects (Salgado et al., 2014; Souto et al., 2015). However, randomized controlled trials found no adverse changes in lipid profiles after up to 24 months of tofacitinib treatment (Kuo et al., 2018; Sands et al., 2021). Given the increased risks of cardiovascular and other diseases linked to dyslipidemia (Zweifler et al., 2011; Zysset et al., 2016; Zuzda et al., 2022), clarifying the real-world effects of these widely used RA medications on blood lipids is clinically important.

Since most evidence on the comparative lipid effects of tofacitinib *versus* adalimumab comes from literature reviews and randomized controlled trials rather than large-scale observational data, this retrospective cohort study aimed to compare dyslipidemia incidence with tofacitinib *versus* adalimumab treatment using the large-scale TriNetX electronic health records database.

## 2 Materials and methods

1) Study Design and Data Source

This study utilized de-identified electronic health records data for over 75 million patients across the TriNetX network, which represents numerous integrated delivery networks, hospitals, and administrative claims data across the United States (Yousaf et al., 2020; Raiker et al., 2021; Zhu et al., 2023). This real-world evidence source contains demographic details, diagnoses, procedures, medications, laboratory tests, and clinical notes restructured into a common format. The study period spanned January 2018 through December 2020.

2) Ethical Statements

The TriNetX Analytics Network is compliant with the Health Insurance Portability and Accountability Act (HIPAA), the US federal law, which protects the privacy and security of healthcare data, and any additional data privacy regulations applicable to the contributing HCO. TriNetX is certified to the ISO 27001:2013 standard and maintains an Information Security Management System (ISMS) to ensure the protection of the healthcare data it has access to and to meet the requirements of the HIPAA Security Rule. The TriNetX Analytics Network was granted a waiver by the Western Institutional Review Board (WIRB) since it solely used aggregated counts and statistical summaries of de-identified data. Furthermore, the utilization of TriNetX for this study received approval from the Institutional Review Board of Chung Shan Medical University Hospital (CSMUH No: CS2-21176).

3) Cohort Selection

**FIGURE 1:**
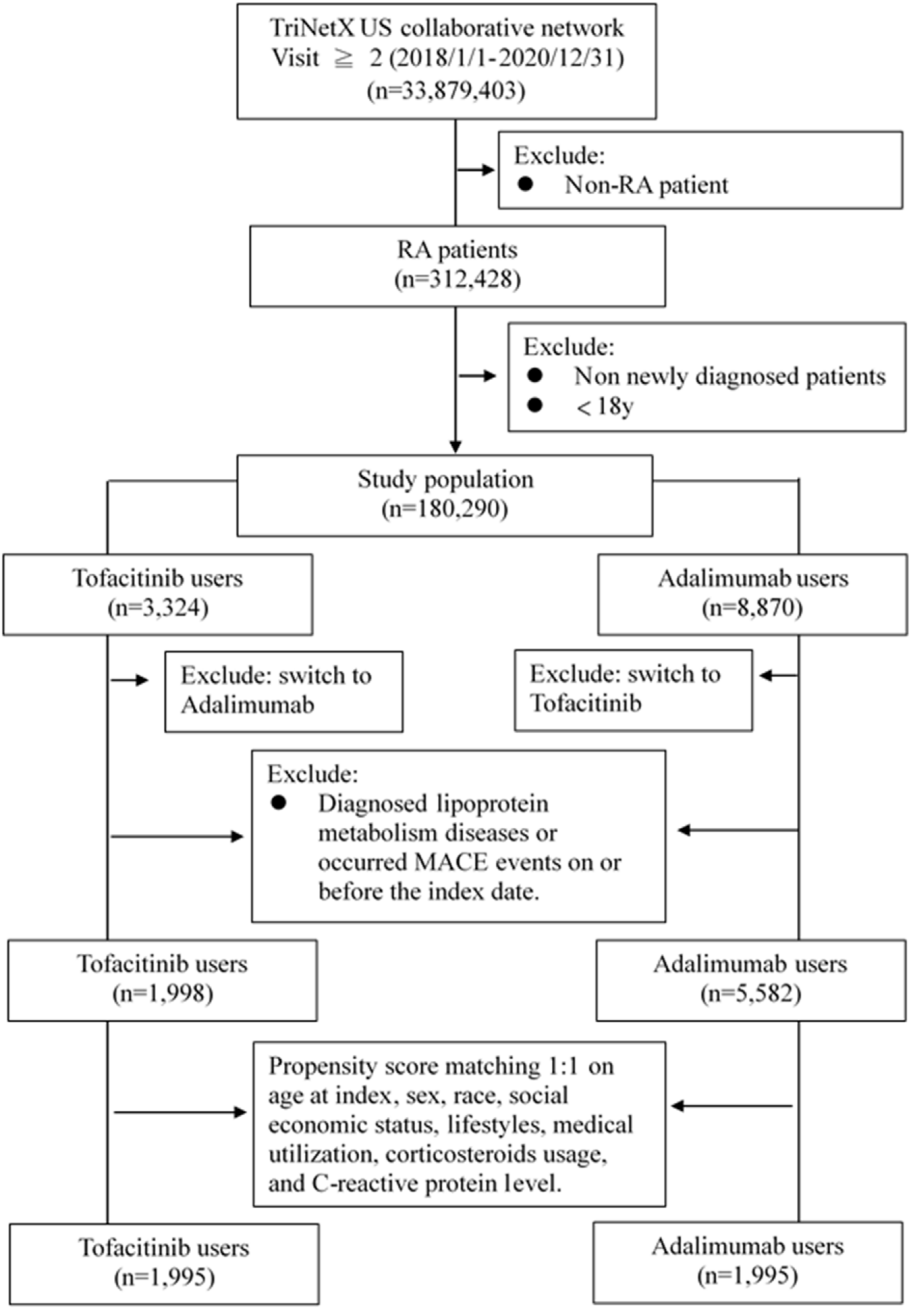
Flow chart of selection.

Adults newly diagnosed with RA between January 2018-December 2020 were included if treated with at least two doses of tofacitinib or adalimumab, without prior diagnoses of dyslipidemia or major adverse cardiac events (MACE). International Classification of Diseases, 10th Revision (ICD-10) codes were used to identify RA (M05-06), dyslipidemia (E78) and related comorbidities. RxNorm codes identified tofacitinib (1,357,536) and adalimumab (327,361) exposure. Patients were assigned to either the tofacitinib or adalimumab cohort based on which medication was received first after RA diagnosis. To balance baseline characteristics, propensity score matching at 1:1 ratio was performed for demographics, lifestyle factors, comorbid conditions, healthcare utilization, corticosteroids usage, and C-reactive protein level (proxy to classify the severity of RA). The participant screening flowchart for this study is shown in Figure 1.

4) Outcomes

The primary outcome was new diagnosis of dyslipidemia (ICD-10 E78) within 3 years of follow-up, including specific subtypes. Secondary outcomes assessed were all-cause mortality and MACE including acute coronary syndrome/unstable angina (ICD-10 I20.0), myocardial infarction (I21-I23), acute ischemic heart disease (I24), stroke (I60–I69, G45), heart failure (I50, I97.1, I11.0), cardiac shock/cardiac arrest (R57.0, I46), or coronary revascularization (coronary artery bypass grafting, defined by current procedural terminology, CPT code 33510–33536), and percutaneous coronary intervention (CPT code 92920–93572). All-cause mortality and MACE were evaluated over the 3-year follow-up period.

5) Other Covariates

Other covariates included: 1) demographic variables (age, sex, race, and potential health hazards related to socioeconomic and psychosocial circumstances); 2) lifestyle factors (tobacco use [ICD-10 Z72.0/Z87.891], nicotine dependence [ICD-10 F17], alcohol-related diseases [ICD-10 F10]); 3) medical utilisation (office or other outpatient services [CPT 1013626], preventive medicine services [CPT 1013829], emergency department services [CPT 1013711], or hospital inpatient services [CPT 1013659]); 4) comorbidities (hypertensive diseases [ICD-10 I10–16], atherosclerosis [ICD-10 I70], diabetes mellitus [ICD-10 E8–E13], obesity [ICD-10 E66], dyslipidaemia [ICD-10 E78.5], depression [ICD-10 F32], anxiety, traumatic past experiences, stress [ICD-10 F40–F48], sleep disorders [ICD-10 G47], chronic lower respiratory disease [ICD-10 J40–J47], non-infective enteritis and colitis [ICD-10 K50–K52], liver disease [ICD-10 K70–K77], systemic lupus erythematosus [ICD-10 M32], chronic kidney disease [ICD-10 N18], cancers [ICD-10 C00–C96], and blood diseases and disorders involving immune mechanisms [ICD-10 D50–D89]); 5) medication (anti-inflammatory and antirheumatic products, non-steroids [Anatomic Therapeutic Chemical (ATC) code M01A], corticosteroids for systemic use [ATC H02], statin [ATC C10AA], and other disease-modifying antirheumatic drugs [sulfasalazine (A07EC01), minocycline (J01AA08), cyclophosphamide (L01AA01), methotrexate (L01BA01), cyclosporine (L04AA01), leflunomide (L04AA13), azathioprine (L04AX01), methotrexate (L04AX03), and hydroxychloroquine (P01BA02)]); and 6) laboratory values (body weight, C-reactive protein in serum, body mass index (BMI), cholesterol; serum or plasma high-density lipoprotein cholesterol; serum or plasma low-density lipoprotein cholesterol; or serum, plasma, or blood triglyceride.

6) Statistical Analysis

Continuous variables were reported as means with standard deviations and categorical variables as counts with percentages. Propensity score matching quality was assessed by calculating absolute standardized differences, with <0.1 indicating good balance. Cox proportional hazards regression modeled time-to-event outcomes in the matched sample, generating hazard ratios (HRs) with 95% confidence intervals (CIs). Survival curves compared dyslipidemia incidence by treatment arm. Subgroup analyses evaluated dyslipidemia risks by sex, age, race, cholesterol, and BMI level. Sensitivity analyses were also conducted to examine the robustness of the results by modifying the initiation time of follow-up.

## 3 Results

1) Study Population and Baseline Characteristics

This study included 7,580 propensity score matched RA patients, with 1998 in the tofacitinib cohort and 5,582 in the adalimumab cohort (Table 1). The mean age was 51.7 and 48.7 years in the tofacitinib and adalimumab groups, respectively. Most patients were female in both treatment arms (78.5% tofacitinib vs. 71.1% adalimumab). After matching, the cohorts were well balanced on demographic factors, with absolute standardized differences <0.1.

**TABLE 1:** Baseline characteristics of study subjects (before and after matching).

Variables	Before PSM			After PSM		
	Tofacitinib users (n = 1,998)	Adalimumab users (n = 5,582)	SMD	Tofacitinib users (n = 1,995)	Adalimumab users (n = 1,995)	SMD
Age at Index						
Mean ± SD	51.7 ± 13.7	48.7 ± 14.1	**0.210**	51.6 ± 13.7	51.2 ± 13.8	0.028
Sex, n (%)						
Female	1,568 (78.5)	3,971 (71.1)	**0.170**	1,565 (78.4)	1,568 (78.6)	0.004
Male	355 (17.8)	1,382 (24.8)	**0.171**	355 (17.8)	350 (17.5)	0.007
Unknown Gender	75 (3.8)	229 (4.1)	0.018	75 (3.8)	77 (3.9)	0.005
Race, n (%)						
White	1,412 (70.7)	3,764 (67.4)	0.070	1,411 (70.7)	1,439 (72.1)	0.031
Black or African American	196 (9.8)	668 (12.0)	0.069	196 (9.8)	182 (9.1)	0.024
Asian	39 (2.0)	130 (2.3)	0.026	39 (2.0)	31 (1.6)	0.031
American Indian or Alaska Native	10 (0.5)	48 (0.9)	0.044	10 (0.5)	10 (0.5)	0.000
Native Hawaiian or Other Pacific Islander	10 (0.5)	10 (0.2)	0.055	10 (0.5)	10 (0.5)	0.000
Other Race	77 (3.9)	254 (4.6)	0.035	77 (3.9)	77 (3.9)	0.000
Unknown Race	261 (13.1)	710 (12.7)	0.010	259 (13.0)	253 (12.7)	0.009
Social economic status, n (%)						
Persons with potential health hazards related to socioeconomic and psychosocial circumstances	10 (0.5)	46 (0.8)	0.040	10 (0.5)	10 (0.5)	0.000
Lifestyles, n (%)						
Nicotine dependence	95 (4.8)	345 (6.2)	0.063	95 (4.8)	76 (3.8)	0.047
Personal history of nicotine dependence	56 (2.8)	203 (3.6)	0.047	56 (2.8)	48 (2.4)	0.025
Tobacco use	14 (0.7)	93 (1.7)	0.089	14 (0.7)	17 (0.9)	0.017
Alcohol related disorders	10 (0.5)	40 (0.7)	0.028	10 (0.5)	10 (0.5)	0.000
Medical utilization, n (%)						
Office or Other Outpatient Services	1,123 (56.2)	3,171 (56.8)	0.012	1,120 (56.1)	1,102 (55.2)	0.018
Emergency Department Services	133 (6.7)	520 (9.3)	0.098	133 (6.7)	125 (6.3)	0.016
Preventive Medicine Services	102 (5.1)	410 (7.3)	0.093	102 (5.1)	94 (4.7)	0.019
Hospital Inpatient and Observation Care Services	47 (2.4)	166 (3.0)	0.039	47 (2.4)	41 (2.1)	0.020
Comorbidities, n (%)						
Diseases of the blood and blood-forming organs and certain disorders involving the immune mechanism	293 (14.7)	789 (14.1)	0.015	293 (14.7)	271 (13.6)	0.032
Hypertensive diseases	236 (11.8)	621 (11.1)	0.022	235 (11.8)	205 (10.3)	0.048
Anxiety, dissociative, stress-related, somatoform and other nonpsychotic mental disorders	149 (7.5)	541 (9.7)	0.080	149 (7.5)	167 (8.4)	0.033
Overweight and obesity	131 (6.6)	413 (7.4)	0.033	131 (6.6)	151 (7.6)	0.039
Sleep disorders	130 (6.5)	371 (6.6)	0.006	129 (6.5)	124 (6.2)	0.010
Depressive episode	115 (5.8)	346 (6.2)	0.019	115 (5.8)	113 (5.7)	0.004
Chronic lower respiratory diseases	113 (5.7)	431 (7.7)	0.083	113 (5.7)	136 (6.8)	0.048
Diabetes mellitus	86 (4.3)	214 (3.8)	0.024	86 (4.3)	75 (3.8)	0.028
Noninfective enteritis and colitis	83 (4.2)	328 (5.9)	0.079	83 (4.2)	109 (5.5)	0.061
Systemic lupus erythematosus (SLE)	53 (2.7)	74 (1.3)	0.095	53 (2.7)	25 (1.3)	**0.102**
Diseases of liver	50 (2.5)	145 (2.6)	0.006	50 (2.5)	49 (2.5)	0.003
Chronic kidney disease (CKD)	24 (1.2)	58 (1.0)	0.015	24 (1.2)	16 (0.8)	0.040
Atherosclerosis	10 (0.5)	28 (0.5)	0.000	10 (0.5)	10 (0.5)	0.000
Melanoma and other malignant neoplasms of skin	10 (0.5)	25 (0.4)	0.008	10 (0.5)	10 (0.5)	0.000
Malignant neoplasms of thyroid and other endocrine glands	10 (0.5)	21 (0.4)	0.019	10 (0.5)	10 (0.5)	0.000
Malignant neoplasms of breast	10 (0.5)	20 (0.4)	0.022	10 (0.5)	10 (0.5)	0.000
Malignant neoplasms of ill-defined, other secondary and unspecified sites	10 (0.5)	13 (0.2)	0.044	10 (0.5)	10 (0.5)	0.000
Malignant neoplasms of lymphoid, hematopoietic and related tissue	10 (0.5)	13 (0.2)	0.044	10 (0.5)	10 (0.5)	0.000
Malignant neoplasms of digestive organs	10 (0.5)	10 (0.2)	0.055	10 (0.5)	10 (0.5)	0.000
Malignant neoplasms of respiratory and intrathoracic organs	10 (0.5)	10 (0.2)	0.055	10 (0.5)	10 (0.5)	0.000
Malignant neoplasms of female genital organs	10 (0.5)	10 (0.2)	0.055	10 (0.5)	10 (0.5)	0.000
Malignant neoplasms of urinary tract	10 (0.5)	10 (0.2)	0.055	10 (0.5)	10 (0.5)	0.000
Malignant neoplasms of lip, oral cavity and pharynx	0 (0.0)	10 (0.2)	0.060	0 (0.0)	10 (0.2)	0.100
Malignant neoplasms of bone and articular cartilage	0 (0.0)	10 (0.2)	0.060	0 (0.0)	0 (0.0)	NA
Malignant neoplasms of mesothelial and soft tissue	0 (0.0)	10 (0.2)	0.060	0 (0.0)	10 (0.2)	0.100
Malignant neoplasms of male genital organs	0 (0.0)	10 (0.2)	0.060	0 (0.0)	10 (0.2)	0.100
Malignant neoplasms of eye, brain and other parts of central nervous system	0 (0.0)	10 (0.2)	0.060	0 (0.0)	10 (0.2)	0.100
Medications, n (%)						
Corticosteroids for systemic use	1,078 (54.0)	3,027 (54.2)	0.005	1,075 (53.9)	1,062 (53.2)	0.013
NSAIDs	665 (33.3)	2022 (36.2)	0.062	664 (33.3)	676 (33.9)	0.013
HMG CoA reductase inhibitors	65 (3.3)	208 (3.7)	0.026	64 (3.2)	75 (3.8)	0.030
Other DMARDs						
methotrexate	646 (32.3)	2,177 (39.0)	**0.140**	644 (32.3)	787 (39.4)	**0.150**
hydroxychloroquine	329 (16.5)	938 (16.8)	0.009	328 (16.4)	340 (17.0)	0.016
leflunomide	203 (10.2)	478 (8.6)	0.055	202 (10.1)	177 (8.9)	0.043
sulfasalazine	114 (5.7)	475 (8.5)	**0.109**	114 (5.7)	158 (7.9)	0.088
azathioprine	38 (1.9)	71 (1.3)	0.050	38 (1.9)	29 (1.5)	0.035
cyclosporine	24 (1.2)	43 (0.8)	0.044	23 (1.2)	14 (0.7)	0.047
minocycline	10 (0.5)	19 (0.3)	0.025	10 (0.5)	10 (0.5)	0.000
cyclophosphamide	10 (0.5)	10 (0.2)	0.055	10 (0.5)	0 (0.0)	0.100
Laboratory, n (%)						
Body weight (lb)						
<150	323 (16.2)	877 (15.7)	0.012	322 (16.1)	291 (14.6)	0.043
150–200	528 (26.4)	1,396 (25.0)	0.032	527 (26.4)	471 (23.6)	0.065
≧200	338 (16.9)	1,244 (22.3)	**0.136**	338 (16.9)	421 (21.1)	**0.106**
C-reactive protein in Serum, Plasma or Blood (mg/L)						
<1	228 (11.4)	630 (11.3)	0.004	227 (11.4)	196 (9.8)	0.050
1–3	207 (10.4)	656 (11.8)	0.044	207 (10.4)	184 (9.2)	0.039
≧3	560 (28.0)	1739 (31.2)	0.069	560 (28.1)	555 (27.8)	0.006
Body Mass Index (BMI, kg/m (Li et al., 2023))						
<30	376 (18.8)	1,104 (19.8)	0.024	375 (18.8)	365 (18.3)	0.013
30–35	188 (9.4)	553 (9.9)	0.017	188 (9.4)	204 (10.2)	0.027
≧35	149 (7.5)	474 (8.5)	0.038	149 (7.5)	169 (8.5)	0.037
Triglyceride, ≧150 mg/dL	77 (3.9)	146 (2.6)	0.070	77 (3.9)	47 (2.4)	0.087
Total Cholesterol, ≧200 mg/dL	97 (4.9)	216 (3.9)	0.048	97 (4.9)	79 (4.0)	0.044
Cholesterol in HDL, <40 mg/dL	56 (2.8)	166 (3.0)	0.010	56 (2.8)	46 (2.3)	0.032
Cholesterol in LDL, ≧130 mg/dL	49 (2.5)	133 (2.4)	0.005	49 (2.5)	41 (2.1)	0.027

Note: Bold font represents a standardized difference was more than 0.1.

If the patient is less or equal to 10, results show the count as 10.

SD: Standard deviation. SMD: standardized mean difference, NA: Not applicable. NSAIDs: Anti-inflammatory and anti-rheumatic products, non-steroids. A Propensity score matching was performed on age at index, sex, race, social economic status, lifestyles, medical utilization, corticosteroids usage, and C-reactive protein level.

At baseline, methotrexate and sulfasalazine were more commonly co-prescribed in the adalimumab group compared to tofacitinib (39.0% vs. 32.3% and 8.5% vs. 5.7%, respectively). These differences were small in magnitude after propensity score matching.

2) Dyslipidemia Risk

**FIGURE 2:**
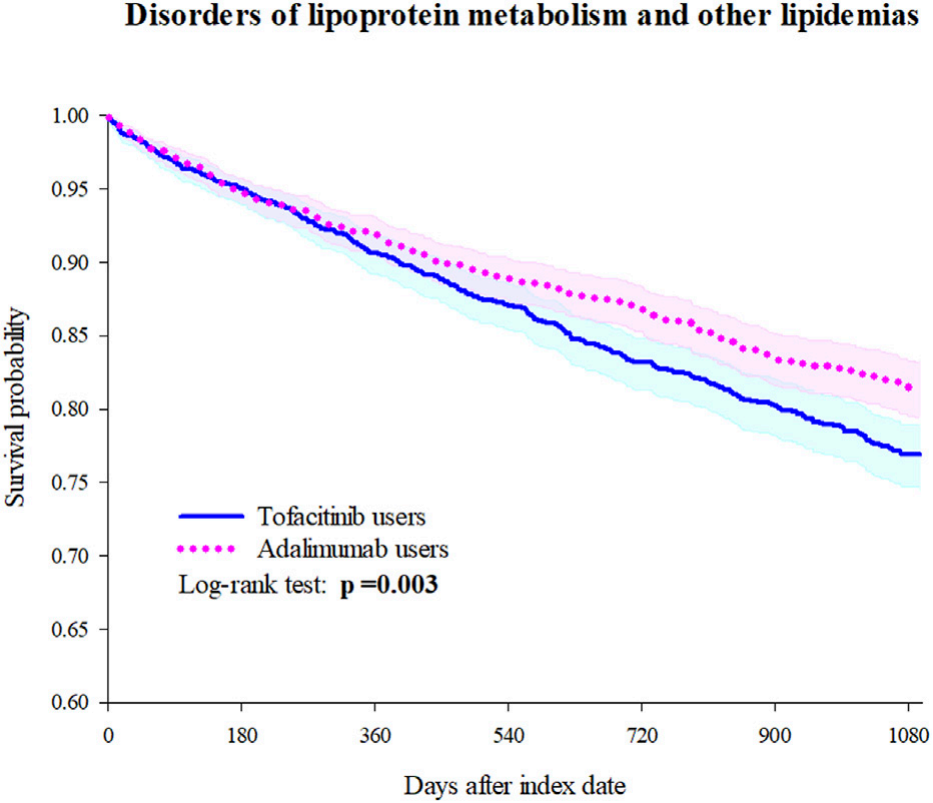
Kaplan-Meier curve of lipidemia disorders incidence.

**FIGURE 3:**
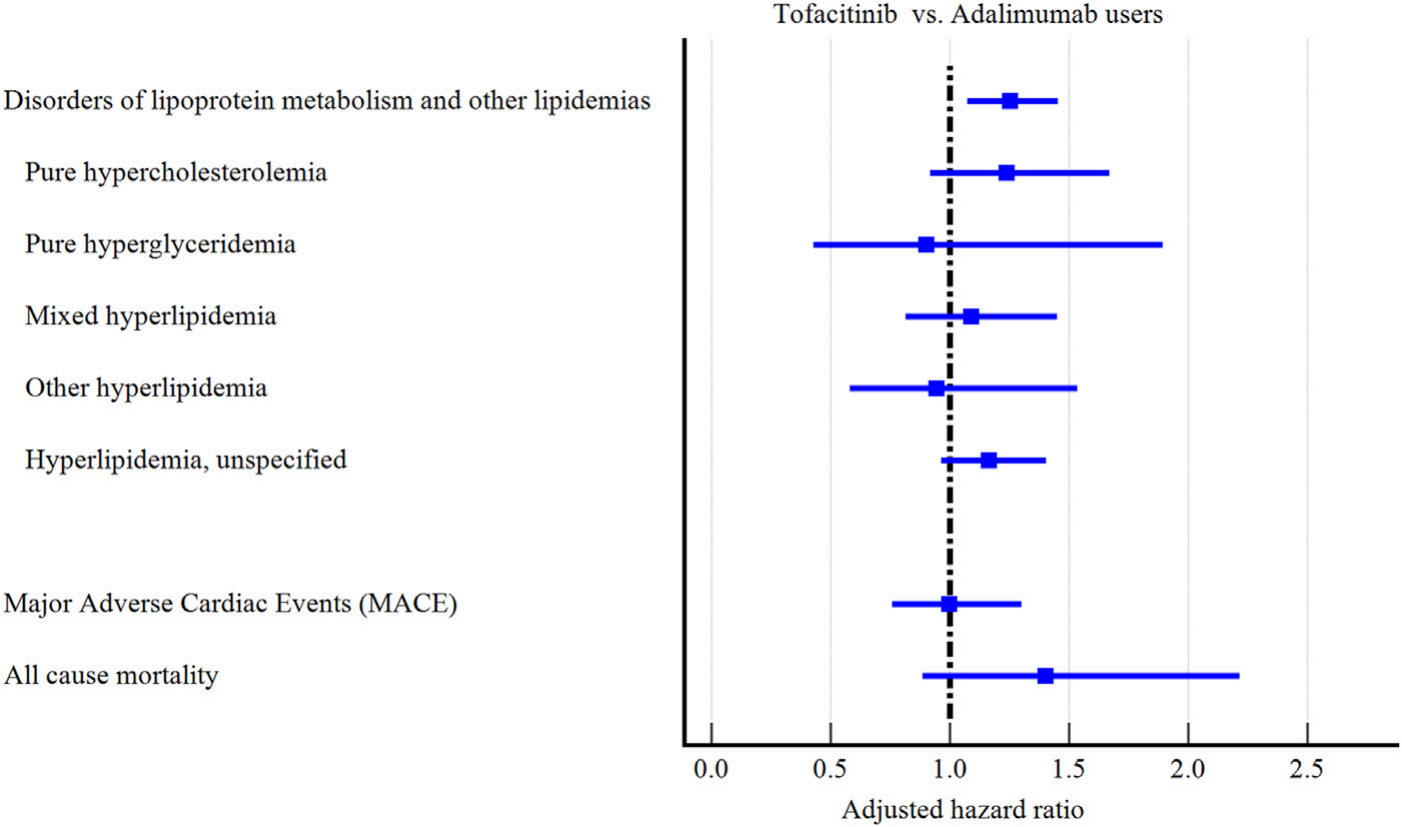
Forest plot of outcomes.

Tofacitinib use was associated with a higher 3-year risk of dyslipidemia *versus* adalimumab (Figure 2; Figure 3; Table 2). The adjusted hazard ratio (HR) for overall dyslipidemia disorders was 1.250 (95% CI 1.076–1.453) in tofacitinib users compared to adalimumab. No significant differences occurred between matched cohorts for major adverse cardiac events (MACE) (HR 0.995 [0.760–1.303]) or mortality (HR 1.402 [0.887–2.215]). After adjusting for various follow-up periods, the dyslipidemia risk associated with tofacitinib use increased over time, while MACE and mortality risks remained comparable to adalimumab (Supplementary Tables S1, S2).

**TABLE 2:** Risk of outcome (1 day–3 years).

Outcomes	Patients with outcome		Adjusted hazard ratio (95% CI)^a^
	Tofacitinib users (n = 1995)	Adalimumab users (n = 1995)	
Disorders of lipoprotein metabolism and other lipidemias	372	315	**1.250 (1.076–1.453)**
Pure hypercholesterolemia	94	79	1.239 (0.918–1.671)
Pure hyperglyceridemia	13	15	0.900 (0.428–1.892)
Mixed hyperlipidemia	95	91	1.089 (0.817–1.452)
Hyperchylomicronemia	0	0	NA
Other hyperlipidemia	31	34	0.944 (0.580–1.535)
Hyperlipidemia, unspecified	228	206	1.163 (0.963–1.404)
Lipoprotein deficiency	0	10	NA
Disorders of bile acid and cholesterol metabolism	0	0	NA
Other disorders of lipoprotein metabolism	10	0	NA
Disorder of lipoprotein metabolism, unspecified	0	10	NA
Major Adverse Cardiac Events (MACE)	104	108	0.995 (0.760–1.303)
All-cause mortality	43	32	1.402 (0.887–2.215)

Note: CI: Confidence interval. NA: not applicable. Bolded value is an important reference for us to draw this conclusion.

In subgroup analyses, both men and women and young patients (18–40 years old) treated with tofacitinib faced the highest dyslipidemia risks compared to those taking adalimumab (HR:1.404, 1.212, and 2.504, respectively, Supplementary Tables S3, S4). Dyslipidemia risk was higher in tofacitinib users compared to adalimumab user among White (HR:1.204 [1.009–1.436] and Black patients (1.218 [0.751–1.976] (Supplementary Table S5). Unexpectedly, elevated baseline cholesterol or BMI status did not clearly amplify the lipid abnormalities associated with tofacitinib (Supplementary Tables S6, S7).

After modified the initiation time of follow-up (start from 2 months, 12 months, 24 months after the index date and followed for 3 years), the dyslipidemia risk consistently associated with tofacitinib, while MACE and mortality risks remained similar to adalimumab (Supplementary Table S8).

## 4 Discussion

This large real-world study of over 7,500 well-matched RA patients aligns with prior evidence that tofacitinib therapy appears to increase the risk of dyslipidemia more than adalimumab (Salgado et al., 2014; Souto et al., 2015; Charles-Schoeman et al., 2016a; Charles-Schoeman et al., 2016b). Proposed mechanisms relate to tofacitinib decreasing inflammation-induced lipid clearance and cholesterol ester catabolism, which are otherwise accelerated in active RA (Charles-Schoeman et al., 2015; Pérez-Baos et al., 2017). Reassuringly, the higher cholesterol levels associated with tofacitinib did not clearly translate to increased MACE compared to adalimumab over 3-year follow-up (Kume et al., 2017).

The findings that women and young adults may warrant closer monitoring for tofacitinib-associated dyslipidemia could inform more tailored RA treatment approaches. No differences in lipid response to tofacitinib *versus* adalimumab were observed by race, contrasting with some cardiovascular studies showing higher event risks in minorities (Khosrow-Khavar et al., 2022; Kristensen et al., 2023a; Kristensen et al., 2023b; Schreiber et al., 2023).

This study was limited by potential misclassification bias, lack of treatment dose-response data, and inability to make conclusions about long-term MACE risks. Additionally, we were unable to surmount certain limitations of the study design, such as the challenge of detecting rare adverse events in small population groups or those with a delayed onset. Furthermore, we employed ICD10 codes to define the disease diagnoses and utilized ATC codes or RxNorm codes on at least two occasions to delineate the prescription of adalimumab or tofacitinib. Within the TriNetX system, we were unable to ascertain whether the diagnosis was rendered by any medical practitioners or specifically by rheumatologists. According to Kim et al. (2011), the Positive Predictive Values (PPVs) were 55.7% for at least two claims coded for RA, 65.5% for at least three claims for RA, and 66.7% for at least two rheumatology claims for RA. The PPVs of these algorithms in patients with at least one DMARD prescription rose to 86.2%–88.9% (Kim et al., 2011). In other words, the accuracy could be substantially enhanced with the incorporation of the drug code. Moreover, due to the constraints of the database platform, we were unable to illustrate the evolution in the utilization of OCS OVER TIME and could only offer data on whether or not OCS was employed in the year preceding the index date for two groups of cases.

These results add to the evidence base around the dyslipidemic effects of RA medications. While tofacitinib seems to have worse lipid profiles than adalimumab, especially in women and young patients, the real-world cardiovascular significance is uncertain. Further research should investigate whether dyslipidemia monitoring and preferential use of adalimumab over tofacitinib in certain higher-risk demographics can improve long-term cardiovascular outcomes. Cost-benefit analysis may also inform RA treatment decisions considering dyslipidemia risks. In conclusion, this study provides clinically useful real-world data to guide management of dyslipidemia as an important potential adverse effect of tofacitinib and adalimumab.

## 5 Conclusion

In this large real-world cohort of patients newly diagnosed with RA, tofacitinib use might be associated with a greater risk of dyslipidemia compared to adalimumab over 3 years, both in men and women and young adults. These observational findings can inform dyslipidemia monitoring and selective use of tofacitinib *versus* adalimumab to potentially mitigate lipid abnormalities in certain higher-risk RA populations. Further research should investigate the long-term cardiovascular safety of tofacitinib given its lipid effects.

## Data Availability

The raw data supporting the conclusion of this article will be made available by the authors, without undue reservation.
