# Supplementary material for: Risk of dyslipidemia and major adverse cardiac events with tofacitinib versus adalimumab in rheumatoid arthritis: a real-world cohort study from 7580 patients
Source: Front Pharmacol. 2024 May 31;15:1370661. doi: 10.3389/fphar.2024.1370661 (PMC11177090; doi:10.3389/fphar.2024.1370661)
Supplement: Supplementary file 1 [file Table1.docx]

Supplementary table S1. Risk of outcomes (1 day to 3 years)_adjusted different variables

| **Outcomes**  (Tofacitinib vs. Adalimumab users) | **Hazard ratio (95% CI)** | | | |
| --- | --- | --- | --- | --- |
|  | **Model 1^a^** | **Model 2^b^** | **Model 3^c^** | **Model 4^d^** |
| **Disorders of lipoprotein metabolism/lipidemias** | **1.328 (1.175-1.501)** | **1.265 (1.088-1.471)** | **1.250 (1.076-1.453)** | **1.210 (1.041-1.406)** |
| Pure hypercholesterolemia | **1.502 (1.171-1.926)** | **1.525 (1.110-2.095)** | 1.239 (0.918-1.671) | 1.343 (0.989-1.823) |
| Pure hyperglyceridemia | 0.854 (0.460-1.586) | 0.961 (0.452-2.045) | 0.900 (0.428-1.892) | 0.614 (0.309-1.218) |
| Mixed hyperlipidemia | 1.234 (0.971-1.569) | 1.283 (0.950-1.734) | 1.089 (0.817-1.452) | 1.009 (0.759-1.342) |
| Hyperchylomicronemia | NA | NA | NA | NA |
| Other hyperlipidemia | 1.148 (0.757-1.741) | 0.946 (0.581-1.539) | 0.944 (0.580-1.535) | 1.038 (0.631-1.707) |
| Hyperlipidemia, unspecified | **1.182 (1.013-1.378)** | 1.146 (0.949-1.383) | 1.163 (0.963-1.404) | 1.158 (0.958-1.399) |
| Lipoprotein deficiency | NA | NA | NA | NA |
| Disorders of bile acid and cholesterol metabolism | NA | NA | NA | NA |
| Other disorders of lipoprotein metabolism | 8.591 (0.894-82.59) | NA | NA | NA |
| Disorder of lipoprotein metabolism, unspecified | NA | NA | NA | NA |
| **Major Adverse Cardiac Events (MACE)** | 1.127 (0.899-1.414) | 1.044 (0.795-1.371) | 0.995 (0.760-1.303) | 0.920 (0.706-1.198) |
| **All-cause mortality** | 1.440 (0.999-2.075) | 1.276 (0.817-1.994) | 1.402 (0.887-2.215) | 1.290 (0.826-2.016) |

Note: CI: Confidence interval. NA: Not applicable.

a. Crude, before matching.

b. Propensity score matching was performed on age at index, sex and race.

c. Propensity score matching was performed on age at index, sex, race, social economic status, lifestyles, medical utilization, corticosteroids usage, and C-reactive protein level.

d. Propensity score matching was performed on all listed characteristics in table 1.

* Proportionality <0.001.

Supplementary table S2. Risk of outcomes_ different follow-up duration

| **Outcomes**  (Tofacitinib vs. Adalimumab users) | **Adjusted hazard ratio (95% CI)**^a^ | | | |
| --- | --- | --- | --- | --- |
|  | **1 day to 1 year** | **1 day to 2 years** | **1 day to 4 years** | **1 day to 5 years** |
| **Disorders of lipoprotein metabolism/lipidemias** | 1.144 (0.923-1.418) | **1.242 (1.049-1.471)** | **1.286 (1.116-1.483)** | **1.298 (1.129-1.492)** |
| Pure hypercholesterolemia | 1.345 (0.823-2.198) | 1.236 (0.864-1.767) | 1.235 (0.929-1.642) | 1.210 (0.917-1.595) |
| Pure hyperglyceridemia | 0.762 (0.264-2.196) | 1.019 (0.382-2.714) | 0.744 (0.376-1.474) | 0.744 (0.376-1.474) |
| Mixed hyperlipidemia | 1.074 (0.689-1.675) | 1.210 (0.867-1.690) | 1.121 (0.855-1.470) | 1.167 (0.896-1.520) |
| Hyperchylomicronemia | NA | NA | NA | NA |
| Other hyperlipidemia | **0.407 (0.179-0.924)** | 0.649 (0.365-1.152) | 1.031 (0.663-1.603) | 1.154 (0.750-1.776) |
| Hyperlipidemia, unspecified | 1.195 (0.907-1.573) | 1.182 (0.953-1.467) | **1.257 (1.055-1.498)** | **1.281 (1.078-1.521)** |
| Lipoprotein deficiency | NA | NA | NA | NA |
| Disorders of bile acid and cholesterol metabolism | NA | NA | NA | NA |
| Other disorders of lipoprotein metabolism | NA | NA | NA | NA |
| Disorder of lipoprotein metabolism, unspecified | NA | NA | NA | NA |
| **Major Adverse Cardiac Events (MACE)** | 0.996 (0.669-1.483) | 1.052 (0.774-1.428) | 1.045 (0.814-1.341) | 1.053 (0.825-1.344) |
| **All-cause mortality** | 1.814 (0.802-4.106) | 1.493 (0.845-2.640) | 1.373 (0.899-2.098) | 1.434 (0.959-2.145) |

Note: CI: Confidence interval. NA: Not applicable.

a. Propensity score matching was performed on age at index, sex, race, social economic status, lifestyles, medical utilization, corticosteroids usage, and C-reactive protein level.

* Proportionality <0.001.

Supplementary table S3. Risk of outcome (1 day to 3 years)_ stratified by sex

| **Outcomes**  (Tofacitinib vs. Adalimumab users) | **Adjusted hazard ratio**  **(95% CI) ^a^** | |
| --- | --- | --- |
|  | **Male**  (n=354 pairs) | **Female**  (n=1565 pairs) |
| **Disorders of lipoprotein metabolism and other lipidemias** | **1.404 (1.008-1.955)** | **1.212 (1.017-1.444)** |
| Pure hypercholesterolemia | 0.806 (0.428-1.518) | **1.450 (1.019-2.063)** |
| Pure hyperglyceridemia | 0.524 (0.131-2.098) | 0.694 (0.312-1.544) |
| Mixed hyperlipidemia | 1.525 (0.770-3.021) | 1.210 (0.851-1.720) |
| Hyperchylomicronemia | NA | NA |
| Other hyperlipidemia | 5.376 (0.627-46.05) | 0.639 (0.355-1.151) |
| Hyperlipidemia, unspecified | **1.707 (1.130-2.579)** | 1.036 (0.829-1.295) |
| Lipoprotein deficiency | NA | NA |
| Disorders of bile acid and cholesterol metabolism | NA | NA |
| Other disorders of lipoprotein metabolism | NA | 2.049 (0.186-22.59) |
| Disorder of lipoprotein metabolism, unspecified | NA | NA |
| **Major Adverse Cardiac Events (MACE)** | 0.963 (0.540-1.718) | 0.912 (0.671-1.240) |
| **All-cause mortality** | 0.728 (0.311-1.703) | 1.646 (0.952-2.844) |

Note: CI: Confidence interval. NA: Not applicable.

^a.^ Propensity score matching was performed on age at index, sex, race, social economic status, lifestyles, medical utilization, corticosteroids usage, and C-reactive protein level.

* Proportionality <0.001.

Supplementary table S4. Risk of outcome (1 day to 3 years)_ stratified by age at index

| **Outcomes**  (Tofacitinib vs. Adalimumab users) | **Adjusted hazard ratio**  **(95% CI) ^a^** | | |
| --- | --- | --- | --- |
|  | **18~40y**  (n=410 pairs) | **41~64y**  (n=1301 pairs) | **≧ 65y**  (n=451 pairs) |
| **Disorders of lipoprotein metabolism and other lipidemias** | **2.504 (1.363-4.600)** | 1.164 (0.972-1.394) | 1.178 (0.933-1.488) |
| Pure hypercholesterolemia | **5.194 (1.492-18.08)** | 1.184 (0.808-1.735) | 1.282 (0.805-2.041) |
| Pure hyperglyceridemia | 1.610 (0.269-9.652) | 0.760 (0.360-1.607) | 0.405 (0.079-2.087) |
| Mixed hyperlipidemia | 2.928 (0.776-11.04) | 1.228 (0.859-1.757) | 1.224 (0.786-1.906) |
| Hyperchylomicronemia | NA | NA | NA |
| Other hyperlipidemia | 2.175 (0.197-24.06) | 1.064 (0.601-1.885) | 1.010 (0.494-2.067) |
| Hyperlipidemia, unspecified | 1.258 (0.581-2.720) | 1.113 (0.886-1.398) | 1.063 (0.806-1.403) |
| Lipoprotein deficiency | NA | NA | NA |
| Disorders of bile acid and cholesterol metabolism | NA | NA | NA |
| Other disorders of lipoprotein metabolism | NA | NA | NA |
| Disorder of lipoprotein metabolism, unspecified | NA | NA | NA |
| **Major Adverse Cardiac Events (MACE)** | 1.203 (0.436-3.318) | 0.838 (0.593-1.183) | 1.128 (0.763-1.667) |
| **All-cause mortality** | 1.645 (0.275-9.852) | 1.730 (0.872-3.435) | 1.341 (0.712-2.526) |

Note: CI: Confidence interval. NA: Not applicable.

^a.^ Propensity score matching was performed on age at index, sex, race, social economic status, lifestyles, medical utilization, corticosteroids usage, and C-reactive protein level.

* Proportionality <0.001.

Supplementary table S5. Risk of outcome (1 day to 3 years)_ stratified by race

| **Outcomes**  (Tofacitinib vs. Adalimumab users) | **Adjusted hazard ratio**  **(95% CI) ^a^** | |
| --- | --- | --- |
|  | **White**  (n=1409 pairs) | **Black or African American**  (n=192 pairs) |
| **Disorders of lipoprotein metabolism and other lipidemias** | **1.204 (1.009-1.436)** | 1.218 (0.751-1.976) |
| Pure hypercholesterolemia | 1.134 (0.803-1.603) | 2.949 (0.939-9.263) |
| Pure hyperglyceridemia | 0.801 (0.364-1.765) | NA |
| Mixed hyperlipidemia | 1.251 (0.860-1.818) | 0.613 (0.241-1.557) |
| Hyperchylomicronemia | NA | NA |
| Other hyperlipidemia | 0.871 (0.464-1.635) | 1.637 (0.273-9.814) |
| Hyperlipidemia, unspecified | 1.154 (0.925-1.440) | 1.324 (0.710-2.470) |
| Lipoprotein deficiency | NA | NA |
| Disorders of bile acid and cholesterol metabolism | NA | NA |
| Other disorders of lipoprotein metabolism | 2.027 (0.184-22.36) | NA |
| Disorder of lipoprotein metabolism, unspecified | NA | NA |
| **Major Adverse Cardiac Events (MACE)** | 0.937 (0.683-1.285) | 1.215 (0.578-2.555) |
| **All-cause mortality** | 1.397 (0.833-2.341) | 1.742 (0.416-7.290) |

Note: CI: Confidence interval. NA: Not applicable.

^a.^ Propensity score matching was performed on age at index, sex, race, social economic status, lifestyles, medical utilization, corticosteroids usage, and C-reactive protein level.

* Proportionality <0.001.

Supplementary table S6. Risk of outcome (1 day to 3 years)_ stratified by cholesterol level

| **Outcomes**  (Tofacitinib vs. Adalimumab users) | **Adjusted hazard ratio**  **(95% CI) ^a^** | |
| --- | --- | --- |
|  | **With high cholesterol^b^**  (n=94 pairs) | **Without high cholesterol^c^**  (n=1413 pairs) |
| **Disorders of lipoprotein metabolism and other lipidemias** | 1.111 (0.677-1.823) | 1.178 (0.953-1.457) |
| Pure hypercholesterolemia | 1.358 (0.413-4.468) | 1.247 (0.819-1.897) |
| Pure hyperglyceridemia | 1.848 (0.308-11.10) | 1.029 (0.257-4.114) |
| Mixed hyperlipidemia | 0.915 (0.332-2.523) | 1.035 (0.652-1.643) |
| Hyperchylomicronemia | NA | NA |
| Other hyperlipidemia | 1.752 (0.292-10.53) | 0.769 (0.364-1.626) |
| Hyperlipidemia, unspecified | 0.867 (0.470-1.600) | 1.121 (0.869-1.447) |
| Lipoprotein deficiency | NA | NA |
| Disorders of bile acid and cholesterol metabolism | NA | NA |
| Other disorders of lipoprotein metabolism | NA | NA |
| Disorder of lipoprotein metabolism, unspecified | NA | NA |
| **Major Adverse Cardiac Events (MACE)** | 0.419 (0.111-1.583) | 1.153 (0.831-1.599) |
| **All-cause mortality** | NA | **1.987 (1.172-3.370)** |

Note: CI: Confidence interval. NA: Not applicable.

1. Propensity score matching was performed on age at index, sex, race, social economic status, lifestyles, medical utilization, corticosteroids usage, and C-reactive protein level.
2. Defined by total cholesterol (≧200 mg/dL) or cholesterol in LDL (≧130 mg/dL) occurred within 1 year on or before the index date.
3. There were no occurrences of total cholesterol (≥200 mg/dL) or LDL cholesterol (≥130 mg/dL) in their electronic health records.

* Proportionality <0.001.

Supplementary table S7. Risk of outcome (1 day to 3 years)_ stratified by BMI level

| **Outcomes**  (Tofacitinib vs. Adalimumab users) | **Adjusted hazard ratio**  **(95% CI) ^a^** | |
| --- | --- | --- |
|  | **BMI < 30 kg/m^2^ ^b^**  (n=391 pairs) | **BMI ≧ 30 kg/m^2^ ^c^**  (n=365 pairs) |
| **Disorders of lipoprotein metabolism and other lipidemias** | 0.746 (0.499-1.115) | 1.056 (0.772-1.445) |
| Pure hypercholesterolemia | 0.783 (0.359-1.706) | 1.163 (0.625-2.164) |
| Pure hyperglyceridemia | NA | 0.426 (0.083-2.202) |
| Mixed hyperlipidemia | 0.749 (0.333-1.687) | 0.692 (0.352-1.362) |
| Hyperchylomicronemia | NA | NA |
| Other hyperlipidemia | 0.493 (0.171-1.420) | 0.919 (0.308-2.739) |
| Hyperlipidemia, unspecified | 0.927 (0.567-1.515) | 1.054 (0.722-1.537) |
| Lipoprotein deficiency | NA | NA |
| Disorders of bile acid and cholesterol metabolism | NA | NA |
| Other disorders of lipoprotein metabolism | NA | NA |
| Disorder of lipoprotein metabolism, unspecified | NA | NA |
| **Major Adverse Cardiac Events (MACE)** | 0.957 (0.497-1.843) | 0.683 (0.388-1.204) |
| **All-cause mortality** | 1.820 (0.754-4.396) | 0.656 (0.214-2.007) |

Note: BMI: Body mass index; CI: Confidence interval. NA: Not applicable.

1. Propensity score matching was performed on age at index, sex, race, social economic status, lifestyles, medical utilization, corticosteroids usage, and C-reactive protein level.
2. BMI did not exceed 30 kg/m^2^, and there were no instances of BMI reaching at least 30 kg/m^2^ within one year prior to or on the index date.
3. BMI at least 30 kg/m^2^ occurred within 1 year on or before the index date.

* Proportionality <0.001.

Supplementary table S8. Sensitivity analyses_ modified the initiation time of follow-up

| **Outcomes**  (Tofacitinib vs. Adalimumab users) | **Adjusted hazard ratio**  **(95% CI) ^a^** | | |
| --- | --- | --- | --- |
|  | **2 months to 38 months** | **12 months to 48 months** | **24 months to 60 months** |
| **Disorders of lipoprotein metabolism and other lipidemias** | **1.311 (1.118-1.538)** | **1.372 (1.136-1.658)** | **1.329 (1.044-1.693)** |
| Pure hypercholesterolemia | 1.249 (0.913-1.708) | 1.161 (0.818-1.650) | 1.114 (0.724-1.714) |
| Pure hyperglyceridemia | 0.746 (0.331-1.679) | 0.793 (0.319-1.973) | 0.552 (0.207-1.472) |
| Mixed hyperlipidemia | 1.058 (0.790-1.419) | 1.148 (0.818-1.611) | 1.020 (0.666-1.563) |
| Hyperchylomicronemia | NA | NA | NA |
| Other hyperlipidemia | 1.112 (0.676-1.830) | 1.593 (0.916-2.773) | **2.620 (1.246-5.508)** |
| Hyperlipidemia, unspecified | **1.241 (1.016-1.517)** | 1.256 (1.000-1.579) | **1.387 (1.047-1.835)** |
| Lipoprotein deficiency | NA | NA | NA |
| Disorders of bile acid and cholesterol metabolism | NA | NA | NA |
| Other disorders of lipoprotein metabolism | NA | NA | NA |
| Disorder of lipoprotein metabolism, unspecified | NA | NA | NA |
| **Major Adverse Cardiac Events (MACE)** | 1.006 (0.753-1.344) | 1.103 (0.799-1.523) | 1.007 (0.674-1.504) |
| **All-cause mortality** | 1.237 (0.778-1.966) | 1.232 (0.742-2.047) | 1.378 (0.780-2.433) |

Note: CI: Confidence interval. NA: Not applicable.

1. Propensity score matching was performed on age at index, sex, race, social economic status, lifestyles, medical utilization, corticosteroids usage, and C-reactive protein level.

* Proportionality <0.001.
